# Using Social Network Analysis to Identify Key Child Care Center Staff for Obesity Prevention Interventions: A Pilot Study

**DOI:** 10.1155/2013/919287

**Published:** 2013-08-05

**Authors:** Jennifer Marks, Lisa M. Barnett, Chad Foulkes, Penelope Hawe, Steven Allender

**Affiliations:** ^1^WHO Collaborating Center for Obesity Prevention, Deakin University, Geelong, VIC 3220, Australia; ^2^School of Health and Social Development, Deakin University, Burwood, VIC 3125, Australia; ^3^Healthy Together Geelong, City of Greater Geelong, Geelong, VIC 3220, Australia; ^4^Population Health and Inequalities Research Center University of Calgary, Calgary, Canada T2N 4N1

## Abstract

*Introduction*. Interest has grown in how systems thinking could be used in obesity prevention. Relationships between key actors, represented by social networks, are an important focus for considering intervention in systems. *Method*. Two long day care centers were selected in which previous obesity prevention programs had been implemented. Measures showed ways in which physical activity and dietary policy are conversations and actions transacted through social networks (interrelationships) within centers, via an eight item closed-ended social network questionnaire. Questionnaire data were collected from (17/20; response rate 85%) long day care center staff. Social network density and centrality statistics were calculated, using UCINET social network software, to examine the role of networks in obesity prevention. *Results*. “Degree” (influence) and “betweeness” (gatekeeper) centrality measures of staff inter-relationships about physical activity, dietary, and policy information identified key players in each center. Network density was similar and high on some relationship networks in both centers but markedly different in others, suggesting that the network tool identified unique center social dynamics. These differences could potentially be the focus of future team capacity building. *Conclusion*. Social network analysis is a feasible and useful method to identify existing obesity prevention networks and key personnel in long day care centers.

## 1. Introduction

Obesity prevention efforts in childhood are needed to arrest the increasing prevalence of obesity [1–3] and its associated health risks [4]. Children's food preferences and eating patterns developed by early exposure to foods [5], along with physical activity and inactivity behaviors, have been shown to track from childhood into adulthood [6]. Regulated center-based childcare (such as long day care) may provide an opportune setting for promoting obesity preventing behaviors in preschool children [7, 8]. A systematic review in 2010 of interventions in childcare settings described one third of the studies as promising in improving children's dietary and/or physical activity behaviors [9], whereas a systematic review in 2011 of interventions in early childhood was critical of current intervention design concluding that social and environmental factors were not given adequate consideration within intervention design and implementation [10].

Those who critique intervention design argue that interventions to tackle childhood obesity must consider a complex system of individual, social, and environmental factors that impact upon eating and activity behaviors [11]. Such a dynamic interrelated system of people, processes, activities, settings, and structures [12, 13] requires a multilevel approach for prevention to be effective [14]. The World Health Organization recognizes the crucial role that people play at each level of a system: as stakeholders, beneficiaries, and mediators, as well as drivers of systems [15]. The integral nature of social interactions is also embedded within the UK National Institute of Health and Clinical Excellence (NICE) core traits of an effective whole system approach to obesity prevention [16]. This NICE review and subsequent guidance proposed a framework for intervention that included capacity building, innovation, working relationships, community engagement, communication, policy action, and leadership [16]. It appears that social structures and relationships within a system could represent a key ingredient for intervention effectiveness, supporting the emerging literature about the importance of identifying and working in partnership with “champions” as strong internal influences and advocates for organizational change [17].

One approach to the identification of social structures and relationships within a system is the use of social network analysis (SNA). SNA describes patterns of social relations and provides a visual tool to help analyze data [18]. SNA has a history of applications within social and behavioral sciences including political systems, community networks, social supports, and group problem solving [19] and can be a useful tool to identify strengths or problems in social structures. SNA is relatively new to health settings although it has been used to: identify key people to improve knowledge sharing efficiency between specialists within hospitals [20]; understand internal/external influences for designing healthcare teams [21]; identify an intervention champion within schools [22]; and identify structural needs for facilitating knowledge transfer between afterschool program teams [23].

The aim of the current study was to determine the feasibility and relevance of SNA for child obesity prevention amongst staff within a long day care setting. To address this aim, this paper asks in relation to dietary and physical activity planning within long day care (LDC) centers:

- What are the relational structures among child care workers that may play a role in obesity prevention practice?
- Can particular players be identified as key to a potential intervention?

## 2. Methods

### 2.1. Sample

We conducted surveys in July 2011 with staff of LDC centers (for children aged 0–5). Informed written consent was obtained from all participants. Ethics approval for this study was granted by the Department of Education and Early Childhood Development (2011_001186) and the relevant University Human Ethics Advisory Group (HEAG-H 63_2011).

The sample was constructed purposefully to be information rich, that is, to provide deep learning and insight [24]. We set out to engage with practitioners who were already aware of and highly sensitized to the opportunities to address obesity in long day care through their involvement in government administered obesity prevention programs. These included Romp & Chomp, which aimed to increase healthy eating and active play in early childhood settings through increasing capacity and local leadership [25], Kids Go For Your Life, which included an active play program for promoting age-related physical activity, and Start Right Eat Right, a training and healthy menu planning program for center directors and cooks. This sampling approach resulted in the inclusion of two centers within one local government area.

Each of the selected centers offered places to 35 children. Each LDC center provided lunch, morning, and afternoon snacks for children and comprised ten staff, including a center director and cook. The directors of the two centers were subsequently contacted for recruitment.

### 2.2. Social Network Questionnaires

A social network questionnaire was developed to articulate and allow quantification of relationships between childcare staff that could potentially influence LDC obesity prevention practice. Eight closed-ended social network questions were constructed to identify: (1) frequency and (2) value (importance) of general information exchange between centers relevant to dietary and activity planning; (3) physical activity information provision and (4) consultation; (5) dietary information provision; (6) decision making and (7) consultation; and (8) network sources of policy information. Questions sought specifically to identify who *provides* dietary, physical activity, and center policy information, who is involved in dietary *decision making* with whom, and who *consults with* whom on information for dietary and physical activity planning to gain an understanding of existing networks. For each question, a list of staff (identified by formal job title within each center) was provided alongside a check box. For most questions, respondents were asked to indicate whether each person was relevant per question, by placing a check if there was a relationship, else leaving blank. For example, questions included: “For each position below, please indicate the type of information (dietary, policy) a person in this position provides for you to do your work,” and “…please indicate who you consult with regarding the amount and type of (food/physical activity) the children (are served/engage in).” Space was also provided for staff to list any key external networks relevant to information sought. For questions on the frequency and value of information, a valued response was required. Information frequency response options ranged from “never” (0), “infrequently” (1), “sometimes” (2), “frequently” (3), to “always” (4). Information value response options were: “not valuable/applicable” (0), “occasionally valuable” (1), “valuable” (2), and “very valuable” (3). All ten staff at each center were invited to complete a social network questionnaire for networks within their center. The questionnaire was piloted with a center director and administrative assistant of a LDC not in the current study. Questionnaires were subsequently modified prior to the study, primarily to reduce questionnaire length.

Written questionnaires were completed by consenting staff at each center, taking approximately ten to twenty minutes, with the researcher based in the staff room during breaks to provide assistance if required. Completed questionnaires were collected by the researcher by the end of each day or returned by mail in prepaid self-addressed envelopes. Questionnaires were analyzed using social network software (UCINET version 6.352) [26].

### 2.3. Social Network Analysis

Density and centralization measures were calculated for each of the eight relationships. Density is the number of ties among staff expressed as a percentage of all possible ties [19]. If all staff had direct ties with all other staff, density would be 100%. Two types of centrality measures were calculated, “degree” and “betweenness” [19]. Degree centrality measures the number of direct links between staff, expressed per individual by number of ties and in total as a percentage of a completely centralized (unequal) network where one person would be at the center of a star like structure with all others in the network connected only to the center player; the higher the percentage, the higher the degree of network centralization [18]. Direction of ties is distinguished by in-degree (receiving ties) and out-degree (sending ties), where high in-degree can indicate prominence and high out-degree can indicate influence. Betweenness centrality refers to the extent that a person lies in-between two other people that would otherwise not be connected [27]. A high “betweenness” score would indicate a person's potential to act as a gatekeeper of information/resources between the people they connect within a particular network. Individual betweenness centrality scores were generated for “provision of information” relationships (activity planning, dietary, and policy) to understand whether key individuals were central to this information. Social network measures were not calculated on results of the question relating to external networks due to this analysis being bound to the internal LDC network “system.” External policy networks were instead described and captured visually.

### 2.4. Presentation of Results

We present results for the frequency and value of information exchanged at each center followed by results for physical activity, dietary, and policy information networks. Social network diagrams are presented for selected results to provide a visual representation to aid description and analysis. Diagrams are described in terms of nodes (network participants) connected or otherwise by lines (ties/relations) that are one directional or two directional (reciprocated) [18]. Nodes (A–J) represent staff (by job title) connected by lines (length not significant) indicating a relationship. For each center, A represents the center director, B–F room-based staff, G–I relieving staff, and J center cook. Centers were physically structured according to children's age groupings (0–2, 2-3, and 3–5), with staff either permanently based in a room with responsibility of one age group (e.g., age 2-3 carer) or rostered to relieve a room based carer (i.e., part-time relieving staff).

## 3. Results 

Questionnaires were completed by 17 of 20 staff (85%): 9 of 10 (90%) staff from LDC center one (LDC1) and 8 of 10 (80%) staff in LDC center two (LDC2). Respondents comprised center directors, cooks, and general staff. Density, degree centralization, and betweenness centralization results for each center and relationship are provided in Table 1.

### 3.1. General Information Exchange Frequency and Value

When asked about the frequency and value of information flow within the LDC setting, the high density scores for both frequency and value relationships for LDC1 (frequency score 86%; value score 85%) and LDC2 (93%; 98%) suggest that staff at both centers felt that “valuable” or “very valuable” information is provided on a very frequent basis relevant to their position at the center (Table 1).

**Figure 1:**
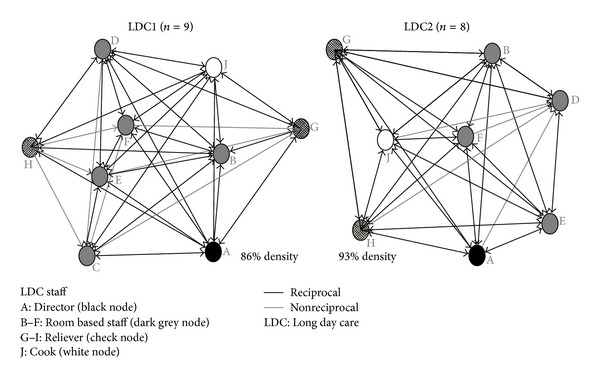
LDC1 and LDC2 information frequency networks.

While both out and in degree centralizations were higher in LDC1 (frequency of information exchange 16%; value of information exchange 17%) than LDC2 (frequency 8%; value 2%), betweenness centralization is low in both centers. The high density and low centrality measures for these relationships suggest that most staff share frequent and valuable information with one another through single relationships rather than through one centralized person or position (Figure 1).

### 3.2. Physical Activity Information Exchange

Individual staff were asked to indicate other individuals who provided them with information to plan physical activity programs. LDC2 had a density score (61%) more than twice that of LDC1 (25%) indicating that more staff in LDC2 were involved and reciprocate physical activity planning information than in LDC1 (Table 1). Density results describe a similar pattern showing that more staff were consulted and/or reciprocated information for planning room based physical activity programs in LDC2 (73%) compared to LDC1 (55%).

**Figure 2:**
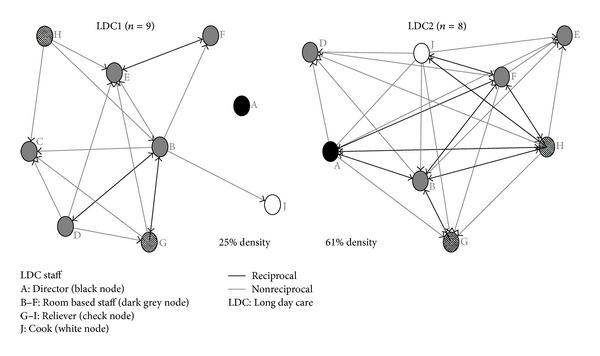
LDC1 and LDC2 physical activity planning information networks.

Figure 2 shows the higher density of connections between staff in LDC2 compared to LDC1. A further difference between centers is the role of the center director. In LDC1, the director (node A) is an isolate, representing that the LDC1 director is not involved in providing information for planning room based physical activity programs at their center. In contrast, the director of LDC2 had relationships with respondents in that center either in the reciprocal provision of information (3 other staff) or a one-directional relationship (4 staff).

LDC1 had higher degree centralization scores for the provision of physical activity information (out-degree: LDC1 56%; LDC2 45%; in-degree: LDC1 42%; LDC 12%) and consultation (out-degree: LDC1 50%; LDC2 31%) indicating more concentration of activity for consultation and sharing information in LDC1 compared to LDC2. Consultation in-degree centralization scores were similarly low at both centers (LDC1 8%; LDC2 14%).

In both centers, betweenness centralization was low (both 13%), which suggests little “gatekeeping” of information between staff (i.e., one staff member “in-between” another for physical activity planning information). Negligible betweenness scores for consulting others indicate that there are no key players at either center for staff to consult regarding physical activity planning. Examining the networks for each individual staff member (Table 2) shows that betweenness centrality scores for “activity planning information” are low for all staff except the age 3–5 carer (staff member responsible for children aged 3–5, node B) at LDC1 (score of 8) and LDC2 (score of 6). This suggests that the age 3–5 carer has a more prominent role in sharing physical activity planning information compared to other staff in that center.

### 3.3. Dietary Information Exchange

**Figure 3:**
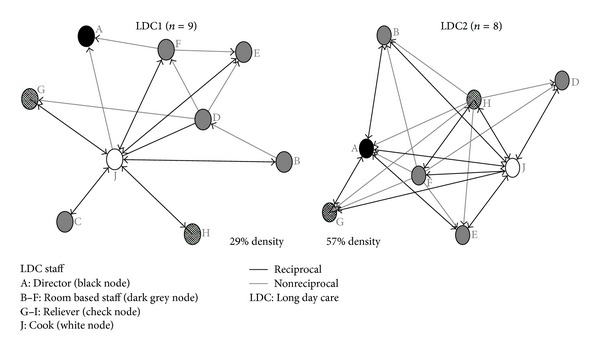
LDC1 and LDC2 dietary information networks.

Staff were asked which other center staff provided them with children's dietary information (e.g., nutrition guidance, menu planning), yielding considerably different results between centers (Table 1). Density scores were almost twice as high for LDC2 (57%) than LDC1 (29%), suggesting less sharing of dietary information between pairs of staff at LDC1 compared to LDC2. Density scores for the level of decision making (LDC1 88%; LDC2 86%) and consultation (e.g., for menu planning) (LDC1 55%; LDC2 73%) suggest that menu planning is a consultative process amongst most staff. Degree centralization scores reveal the role of providing dietary information for staff at the centers as highly centralized: more so for LDC1 (out-degree 80%; in-degree 66%) than LDC2 (out-degree 49%; in-degree 49%). High dietary information betweenness centralization scores (LDC1 73%; LDC2 49%) indicate key staff having a prominent role in sharing of dietary information. Betweenness scores for dietary decision making (LDC1 4%; LDC2 6%) and consultation (LDC1 12%; LDC2 1%) were relatively low by comparison reinforcing the notion that key individuals led the center's around food quality. Individual betweenness scores for the center cooks (node J) were very high in both centers (LDC1 41; LDC2 21) relative to all other staff (LDC1 0–2; LDC2 0–3), indicating that the cook has a prominent role for providing dietary information within the centers (Table 2). Yet the difference in scores between center cooks is considerable, the LDC1 cook having almost twice the score as the cook at LDC2.  Figure 3, providing a visual depiction of dietary information networks, highlights the greater degree of centralization at LDC1 compared to LDC2, shown by the (unequal) star like structure with the cook (node J) quite central.

### 3.4. Policy Information Exchange

Staff were asked to indicate who provides them with policy information relevant to their role from within and external to their individual center. A large difference in density scores indicates more sharing of policy information between staff at LDC2 (68%) compared to LDC1 (31%). The provision of policy information is highly centralized reflected in higher centralization scores, particularly in LDC1 (out-degree 78%; in-degree 50%) compared to LDC2 (out-degree 37%; in-degree 37%). Despite the large difference in density and degree centralization scores between centers, betweenness centralization scores were both similar and relatively high at both centers (LDC1 45%; LDC2 43%). Individual betweenness scores suggest that the leader on policy information differs between centers. In LDC1, the center cook is highly centralized, having a high betweenness score (26%) regarding policy information compared to other staff (0–5%) and the LDC2 cook (0%). In LDC2, the director has a high betweenness score (18%) within a denser more reciprocated network, compared to all other staff at their center (0%) and the LDC1 director (0%).

### 3.5. External Sources of Policy Information

**Figure 4:**
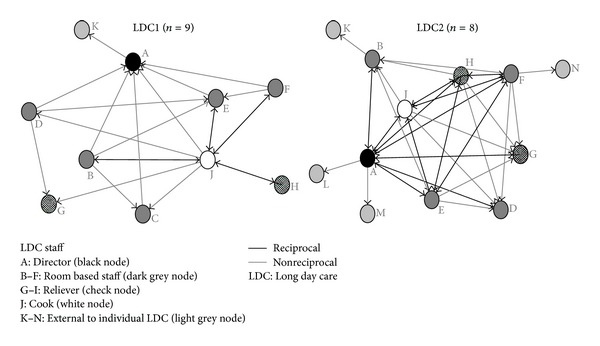
LDC1 and LDC2 policy information networks.

Responses also differed between center staff regarding key external sources of policy information (Figure 4). The regional childcare coordinator (node K) was the only external source identified by LDC1 (by the director), whereas three staff at LDC2 identified four external sources. This included the regional childcare coordinator, identified as an external policy source by a room-based carer relatively new to the center. Two prior intervention coordinators (nodes L & M) were identified by the LDC2 director as continuing to be contacts for sourcing policy information. A fourth source was identified by an internal LDC2 relieving staff member who acknowledged external relieving staff (node N) as their information source.

## 4. Discussion

This study aimed to determine the feasibility and usefulness of SNA for child obesity prevention within LDC by identifying childcare staff networks and key players that could potentially influence LDC obesity prevention practice. Within this pilot of two LDC centers, we found the identification and quantification of internal LDC staff networks provided insight into existing structures primed for obesity prevention practice. General communication networks were similar at both centers, yet distinct differences between specific information networks were also found to exist. This included the identification of key players for future intervention, based on ties within the network rather than formal center job title. One center cook compared to another was notably more central for dietary and associated policy information; one director was not involved in room-based physical activity planning information, whilst the other director was heavily involved in all day-to-day planning operations. SNA provided a method that was easy to administer and find these distinctions, demonstrating the potential for use in future intervention planning within the child care setting.

General information exchange relating to dietary and activity planning within each center was found to be frequent, relevant, and highly reciprocated between staff. These decentralized dense information structures suggest strong potential for effective dissemination of any new information entering the networks. Information exchange (bidirectional), as differentiated from information transfer (unidirectional), is argued to be more effective in communicating health practice and producing action [28].

We found notable differences in management involvement in day-to-day operations between centers, particularly for planning aged-based physical activity programs. Centers within Australia require national childcare quality guidelines [24] to be adopted for planning individual age/development appropriate children's programs. Within the US, although childcare is heavily regulated, physical activity (and dietary) guidelines have been found to vary between states [7]. Few centers in Australia have trained staff or policies in relation to physical activity [29]. Encouraging physical activity by training staff and following written policy are areas where centers can improve obesity prevention best practice [30]. LDC centers within the current study benefited from their prior intervention involvement as this encouraged the incorporation of childcare quality guidelines into center practice through staff training in fundamental movement skills and the design of structured active play programs tailored to each center [25]. This suggests that promoting and implementing physical activity practice in childcare are more than policy, requiring flexibility, training and guidance, and the consideration of differences in management styles and guideline interpretation. SNA provides an easy to use method to identify differences in existing networks. This could be used to tailor future health promotion training and team capacity building to these different social dynamics. In other words, more emphasis on some topics than others in some centers and interrogation of why some people are more or less the “go to” people on particular topics.

The center cook was found to play a key role regarding dietary information. Betweenness centrality revealed one cook as more prominent than the other despite both cooks having similar tenure at their respective centers and both centers previously receiving and implementing the same nutrition intervention. An implication of this finding is the possibility for using SNA within intervention planning to locate strategic personnel as program “champions” for training and disseminating health promotion information [22]. A US study of the relationship between childcare workers knowledge, beliefs, and practices stressed the importance of childcare staff having a role in nutrition education for promoting healthy eating and obesity prevention [31]. It seems childcare staff would be receptive; a UK study found enthusiasm in childcare workers to provide healthy food within centers in need of nutrition policy and staff training [32]. In this sense, the findings of our study are not unique. Cooks and program directors are obvious key players in nutrition policy and practice. But it is unique to start to build metrics around the capacities of centers (operationalized here as the densities of the relationships) and the centrality scores of key people. SNA provided further insight within these key positions, demonstrating that individuals holding the same center job title may not play the same strategic role.

The SNA highlighted different policy information networks between centers where the cook was prominent in one center as a program champion for nutrition. In contrast, the director of the other center demonstrated stronger policy links with external networks. Previous studies of social networks in healthcare teams have suggested that external networks are important for collecting and disseminating information, whilst internal structures are important for knowledge sharing [21]. Applying this perspective to the LDC findings suggests that SNA successfully identified an LDC director having a highly strategic position to promote obesity prevention practice both within the center and the broader early years community.

We also found internal LDC networks with ongoing connections to extended networks as sources of policy information. These included prior intervention coordinators and external relieving staff. A recent multisite afterschool care study found high levels of skill transfer between staff (77%), even in networks with low program connection density (2%), concluding that informal networks are potentially underutilized in this setting [33]. Our findings also suggest that there is potential to explore the extent of external network influence not only as sources of knowledge but also in sharing skills and knowledge between centers. We used SNA to look at existing networks where obesity prevention intervention had previously been implemented and found evidence of strong relational structures and practices supporting best practice in dietary management and physical activity programming within the centers. This suggests that prior intervention was instrumental in the creation of additional external networks (as continuing sources of policy information), promoting obesity prevention practice. A pediatric obesity prevention intervention found the creation of new social networks a critical outcome, highlighting the potential for sharing health behavior knowledge and practice amongst mothers of preschool aged children [34]. Using SNA to identify the creation of new ties, in addition to understanding existing network dynamics, could be imperative for understanding intervention effects.

A limitation of this study was the small sample, restricting generalizability of findings to the two day care centers involved. A study strength was the ease of using social network analysis to identify existing networks and key staff within each LDC. We examined healthy eating and physical activity information networks. This study did not examine the relationship between the networks identified and specific action to prevent obesity nor did it consider outcomes such as children's anthropometric measurement and the association with network structure. Future research should investigate how network structure impacts upon obesogenic/healthy childcare environments and children's health and weight status.

## 5. Conclusion

This study demonstrated the feasibility and relevance of SNA for identifying existing communication networks and strategic staff for promoting obesity prevention practice within the LDC setting. SNA represents a potentially valuable tool for understanding LDC network structures and identifying important players for tailoring intervention planning and building team capacity relevant to each LDC context. 

## Figures and Tables

**Table 1:** Density and centralization scores for each relationship.

Relationship	Density (%)		Degree centralization (%)				Betweenness	
			Out	In	Out	In	centralization (%)	
	LDC1	LDC2	LDC1		LDC2		LDC1	LDC2
Frequency of information	86	93	16	16	8	8	3	2
Value of information	85	98	17	17	2	2	3	<1
Physical activity planning								
Provision of information	25	61	56	42	45	12	13	13
Consultation	55	73	50	8	31	14	0	<1
Dietary (amount and type of food)								
Provision of information	29	57	80	66	49	49	73	49
Decision making	88	86	14	14	16	16	4	6
Consultation	74	82	30	16	20	4	12	1
Policy (internal network)								
Provision of information	31	68	78	50	37	37	45	43

**Table 2:** Individual betweenness centrality scores for “provision of information” networks.

	Job title	Activity planning information		Dietary information		Policy information	
		LDC1	LDC2	LDC1	LDC2	LDC1	LDC2
A	Director	0	0	0	3	0	18
B	Age 3–5 carer	8	6	0	0	0	0
C	Age 2-3 carer	0	*n/a *	0	*n/a *	0	*n/a *
D	Age 2-3 carer	0	0	2	0	0	0
E	Age 0–2 carer	2	0	0	0	5	0
F	Age 0–2 carer	0	2	<1	0	0	0
G	Reliever	0	0	0	0	0	0
H	Reliever	0	2	0	0	0	0
I	Reliever	*n/a *	*n/a *	*n/a *	*n/a *	*n/a *	*n/a *
J	Cook	0	0	41	21	26	0

Mean		1	1	4	3	3	2
SD		2	2	13	7	8	6
